# Our Removal to New York

**Published:** 1881-05

**Authors:** 


### OUR REMOVAL TO NEW YORK.

The June number of the INDEPENDENT PRACTITIONER will be issued from our new quarters, *New York City*. After mature thought and minute investigation, we have reached the conclusion that the best interests of all concerned, demand that we add to our staff a gentleman who will give the journal the advantage of his practical business experience. *We have found the man*, and our facilities at the continent's great business centre, will be first-class in every particular. Our subscribers and advertisers in this and other countries, will find us up to the front. It 's the purpose of the proprietor to make the monthly equal, if not superior to any thing issued in this or any other land, and we hope to add every month many new friends to our list. For the *present*, all mail matter will be received, as formerly, in Baltimore.

